# Prevalence of physical activity levels and perceived benefits of and barriers to physical activity among Jordanian patients with coronary heart disease: A cross-sectional study

**DOI:** 10.3389/fpubh.2022.1041428

**Published:** 2023-01-04

**Authors:** Eman Alsaleh, Faris Baniyasin

**Affiliations:** ^1^School of Nursing, Philadelphia University, Amman, Jordan; ^2^Department of Physiotherapy, Philadelphia University, Amman, Jordan

**Keywords:** physical activity, perceived benefits, perceived barriers, coronary heart disease, prevalence

## Abstract

**Background:**

Many studies published in other countries have identified certain perceived benefits of and barriers to physical activity among patients with coronary heart disease. Nevertheless, there is no data about the issue relating to Jordanian patients with coronary heart disease.

**Objective:**

This study aimed to describe the prevalence of levels of physical activity, the benefits of and barriers to physical activity as perceived by Jordanian patients with coronary heart disease, and the relationship between physical activity and perceived benefits of and barriers to physical activity. In addition, it focused on examining the influence of selected sociodemographic and health characteristics on physical activity and the perceived benefits of and barriers to physical activity.

**Methods:**

A cross-sectional design was performed on a sample of 400 patients with coronary heart disease. They were given a list of perceived benefits of and barriers to physical activity and asked to what extent they disagreed or agreed with each.

**Results:**

Jordanian patients with coronary heart disease perceived various benefits of and barriers to physical activity. Most of these benefits were physiologically related (average mean = 5.7, SD = 0.7). The most substantial barriers to physical activity as perceived by the patients were “feeling anxiety,” “not enough time,” “lack of interest,” “bad weather,” and “feeling of being uncomfortable.” Sociodemographic and health characteristics that significantly influenced perceived barriers to physical activity were age, gender, health perception, chest pain frequency, education, job, caring responsibilities, ability to travel alone, smoking, and previous and current physical activity behavior.

**Conclusion:**

This study demonstrates that patients with coronary heart disease have perceived physiological benefits of physical activity and have perceived motivational, physical health, and environmental barriers to physical activity, which is significant in developing intervention strategies that aim to maximize patients' participation in physical activity and overcome barriers to physical activity.

## Introduction

Physical activity is essential in preventing and treating coronary heart disease (CHD) (1–3). The standard PA recommendation for patients with CHD is ≥150 min of moderate-intensity PA per week (4). Evidence suggests that PA markedly contributes to a decrease in modifiable CHD risk factors such as increased BP and overweight (5–7), which are associated with a decrease in the negative impact of CHD on physical health. Significantly, many reviews have reported a decrease in BP among patients with CHD who participated in the recommended level of PA (8–10). In addition, PA increases PA capacity and improves endothelial function, facilitating coronary blood flow through the vasodilatation mechanism (11). These physiological effects of PA have been shown to lower relapse rates, symptoms, and cardiac ischemia after cardiac events among patients with CHD (12–14). Furthermore, PA has been demonstrated to improve quality of life (QoL) and decrease anxiety and depression among patients with CHD (15, 16), even though it has been documented that the benefit from physical activity among patients with CHD is more significant than that of PA among healthy subjects without CHD (12).

Although the significant benefits of PA are well-known in the treatment of CHD, the vast majority of patients with CHD do not achieve PA following recommendations, even when they engage in a structured PA program or cardiac rehabilitation program (17). The performance of regular PA is considered one of the principal challenges in the success of the secondary prevention regimen of these patients through modifications in their lifestyle habits (17, 18). In Jordan, it was found that only 34.8% of patients with CHD performed regular walking exercises (19).

The contributing factors of low performance and adherence to PA that have been consistently reported in the literature include individual characteristics, such as motivation and self-efficacy. Perceived barriers to PA, such as time and access, and characteristics of PA behavior, such as PA type, intensity, and duration (20). In addition, the lack of PA programs and the referral of patients with CHD to these programs, as well as inaccessible PA programs, are significant reasons for low adherence to PA among patients with CHD (21, 22). Patients with CHD in Jordan do not receive structured secondary prevention or cardiac rehabilitation programs. Usually, these patients are provided with verbal or written advice about their disease and lifestyle modifications, including the performance of PA. This information is usually provided during the hospital stay or after discharge during patients' attendance at the clinic for follow-up care, which could hinder patients from performing PA and may be considered a principal barrier to performing PA. Although many studies have examined the relationships between perceived benefits of and barriers and PA participation in western societies, little information is available about how Jordanian patients with CHD perceive the benefits of and barriers to PA. In addition, how these constructs explain the patients' decision to PA is rarely examined.

The health promotion model (HPM) identifies perceived benefits of and barriers to action as influencing factors for health-promoting behavior, such as participation in PA. In this model, health-promoting behavior is the desired outcome (23, 24). The benefits of PA are defined as “a person's perceptions of positive and enjoyable outcomes of this behavior” (25). PA has perceived benefits that are incorporated into physiological, psychological, social, and body image benefits (26, 27). The motivational value of perceived benefits is based on previous personal experiences or outcomes observed by others. Perceived barriers to PA are linked to individuals' challenges when performing PA as an inconvenience, expense, difficulty, time, physical condition, or environmental barrier. The perceived barriers influence the initiation of a new PA or reduce an individual's commitment and adherence to the current PA. Systematic reviews have demonstrated an inverse relationship between the perception and number of barriers and levels of PA among patients with CHD (28).

The significant aspect of the success of programs and interventions that aim to promote PA in patients with CHD is the recognition of factors that limit participation in these programs (29). Information about the benefits of and barriers to PA perceived by Jordanian patients with CHD can inform nurses and healthcare providers, which helps develop an appropriate individualized approach based on patients' perception of PA benefits of and barriers to maximize patient participation in overcoming barriers to PA. These interventions could significantly assist patients with CHD in obtaining the beneficial effects of PA to improve their health, prevent complications, and thus decrease the mortality of CHD.

This study aimed to describe the prevalence of PA levels in patients with CHD and the benefits of and barriers to PA as perceived by Jordanian patients with CH and describe the relationship between perceived benefits of and barriers to PA and the participation of these patients in PA. Furthermore, it aimed to describe the influence of selected sociodemographic and health characteristics on perceived benefits of and barriers to PA and the participation of PA.

## Methodology

First, approval from the Institutional Review Board at King Abdullah University Hospital (KAUH) and Jordan University Hospital administration to interview the patients was secured. Then, the researcher reviewed patients' files who visited the cardiac clinic between 1 July 2019 and 1 October 2019. Those who met the inclusion criteria were identified and invited to participate in the study. A structured interview technique to collect the required data was used.

The sample size was 400 patients with CHD, determined based on the prevalence of PA among CHD in the previous study by determining 40% at a 95% CI, 5% margin of error and adding 37 patients considering dropouts among participants (30). Two university hospitals in Jordan were included to recruit the participants. The inclusion criteria were patients diagnosed with angina or myocardial infarction (MI) at least 4 months before data collection, who were mentally competent, and who were aged 30–70 years. Exclusion criteria included any physical or psychological condition that affected patients' participation in the study.

### Physical activity

The participants were administered the International Physical Activity Questionnaire (IPAQ), which assesses physical activity status. The instrument asks questions about the performance of 30 min of moderate-intensity activity 5 days per week (150 min per week) or a combination of walking and moderate-intensity activities amounting to a minimum of at least 600 METs-minutes/week. The IPAQ is a reliable and valid tool for assessing PA (reliability: kappa 0.67–0.73; Spearman's rho 0.67–0.81).

### Benefits of and barriers to PA questionnaire

Since there are no clinical tools for the reliable evaluation of perceived benefits of and barriers to PA among patients with CHD, the researcher developed a scale consisting of two lists of perceived benefits of and barriers to PA based on the literature. The first list contains 22 items that reflect PA's physical, psychological, and social benefits. The second list contains 41 items that reflect PA's physical, psychological, social, and environmental barriers. The response for each item was on a 6-point Likert scale ranging from (1) strongly disagree to (6) strongly agree. In addition, two open-ended questions asked participants' opinions about additional benefits of and barriers to PA. Items of perceived benefits and barriers demonstrated a high level of internal consistency; Cronbach's alpha was calculated: 0.87 for perceived benefits of PA and 0.92 for perceived barriers to PA. Also, test-retest reliability for the total instrument was 0.89 for the Exercise Benefits Scale and 0.77 for the Exercise Barriers Scale.

To evaluate the clarity and understandability of the perceived benefits of and barriers to PA, the researcher interviewed ten patients with CHD in the cardiac clinics of both hospitals before starting data collection using structured interviews. The patients were six males and four females. In total, seven patients were diagnosed with MI, and three patients with angina. Results showed that all participants reported that all items were clear, understandable, and at the level of the patient's comprehension.

### Statistical analysis

Descriptive statistical analysis was used to describe the sample. The Pearson correlation was used to describe the relationship between the benefits of and barriers to PA and the continuously measured sociodemographic and health characteristics. The same test was used to describe the relationship between individual and health characteristics, and PA was measured continuously. In addition, the *t*-test, chi-square test, and Fisher's exact test were used to examine differences in perceived benefits of and barriers to PA and individual and health characteristics concerning PA behavior that was measured on a continuous and dichotomous level. A one-way analysis of variance was used to compare the means of two or more independent groups.

## Results

### Demographic and health characteristics of the study

The participants were 400 patients with CHD between 36 and 70 years old, with two-thirds being male. There were no significant differences between the two groups (Tables 1,2).

**Table 1:** Sociodemographic characteristics of the sample (*n* = 400).

**Variables**		**Frequency**	**%**	**Mean**	**(SD)**	**Range**
Age				54.34	(8.94)	36–70
Gender	Male	259	64.7			
	Female	141	35.3			
Marital status	Married	341	85.3			
	Single	40	10			
	Widowed	12	3			
	Divorced	7	1.7			
Living status	With family	350	87.5			
	Alone	50	12.5			
Education level	Illiterate	46	11.5			
	Primary	74	18.5			
	Secondary	100	25.0			
	College	150	37.5			
	Baccalaureate	24	6			
	Graduate studies	6	1.5			
Occupation	Yes	240	60			
	No	100	25			
	Retired	60	15			
Type of Job	Laborers	195	48.7			
	Office work	45	11.3			
	No work	160	40			
Income per month				321	(125)	1–1,500
Caring responsibilities	Yes	211	52.7			
	No	189	47.3			

SD, standard deviation.

**Table 2:** Health characteristics of the sample (*n* = 400).

**Variables**		**Frequency**	**%**	**Mean**	**(SD)**	**Range**
Medical diagnosis	Angina	223	55.7			
	MI	177	44.3			
Treatment	Invasive intervention(PTCA, stent)	121	30.3			
	Open heart surgery	60	15			
	Medications	219	54.7			
Chronic diseases	Yes	188	47.0			
	No	212	53.0			
Diseases type	DM	90	22.5			
	HTN	65	16.2			
	DM and HTN	33	08.3			
PA advice	No advice	165	28.0			
	Weak	160	32.0			
	Moderate	20	30.0			
	Strong	55	10.0			
PA status	Physically active	75	18.7			
	Non physically active	325	81.3			
Perception of health	Excellent	49	12.3			
	Very good	52	13			
	Good	140	35			
	Weak	159	39.7			
Ability to travel alone	Yes	188	47			
	No	212	53			
Chest pain frequency				5	0.77	0–5
Smoking	Yes	180	45			
	No	220	55			

SD, standard deviation; MI, myocardial infarction; PTCA, percutaneous transluminal coronary angioplasty; DM, diabetes mellitus; HTN, hypertension; PA, Physical activity.

### Physical activity prevalence

According to the International Physical Activity Questionnaire (IPAQ), 24% of the patients are classified as physically active. According to IPAQ, participants were recognized as physically active when they met the PA guidelines of 30 min of moderate-intensity activity 5 days/week (150 min/week) or engaged in a combination of walking and moderate-intensity activities for a minimum of at least 600 METs-min/week.

### The influence of patient's characteristics on PA status

It was found that male respondents and laborers were significantly more engaged in PA than females and office workers. Also, there was a statistically significant difference between physically active and non-physically active participants regarding physical activity advice, chest pain frequency, and perception of health. Patients with weak PA advice perceive their health as poor and have more chest pain frequency. They also have low PA levels. However, other factors such as marital status and smoking showed no statistically significant relationship with PA engagement (Tables 3, 4).

**Table 3:** Association between participants' sociodemographic characteristics and physical activity.

	**Physically active**				**Test**	***p***
	**Yes**	**(%)**	**No**	**(%)**		
Age mean (SD)	29.70	8.20	29.04	7.54	– 0.750^†^	0.454
**INCOME PER MONTH**						
**Gender**						
Male	191	47.7	68	17	7.393^!^	0.007^*^
Female	62	15.5	79	19.8		
**Occupation**						
Yes	122	30.5	118	29.5	0.010^!^	0.921
No	88	22	72	18		
**Occupation type**						
Office workers	24	6	21	5.3		0.365
Laborers	112	28	83	20.7		0.002^*^
**Marital status**						
Married	190	47.5	151	37.7	0.327^#^	0.955
Single	22	5.5	18	4.5		
Widow	6	1.5	6	1.6		
Divorce	4	1	3	0.7		
**Smoking**						
No	100	25	120	30	0.155^!^	0.694
Yes	96	24	84	21		
**Living status**						
Alone	22	5.5	28	7		
With family	178	44.5	172	43		
Educational level					0.231	0.623
Illiterate	22	5.5	24	6		
Primary	34	8.5	40	10		
Secondary	52	13	48	12		
College	77	19.2	73	18.3		
Baccalaureate	14	3.5	10	2.5		
Graduate	4	1	2	0.5		
**Ability to travel alone**						
Yes	90	22.5	98	24.5	12.177^#^	0.007^*^
No	108	27	104	26		
Caring responsibilities					0.241^!^	0.324
Yes	108	27	103	25.7		
No	98	24.6	91	22.7		

† Independent *t*-test.

! Chi-square test.

# Fisher's exact test.

* Significant *p*-value of <0.05.

SD, standard deviation.

**Table 4:** Association between participants' health characteristics and physical activity.

	**Physically active**				**Test**	***p***
	**Yes**	**(%)**	**No**	**(%)**		
**Perception of health**						
Excellent	74	18.5	25	6.2	0.189^#^	0.910
Very good	76	19	26	6.5		0.004
Good	24	6	66	16.5		
Weak	27	6.75	82	20.5		
Chest pain frequency mean (SD)	24.80	3.99	26.26	4.34	3.132^†^	0.002^*^
**Chronic diseases**						
Yes	98	24.5	90	22.5	12.177^#^	0.007^*^
No	104	26	108	27		
**PA advice**						
No advice	81	20.2	84	21		
Weak	79	19.7	81	20.4		
Moderate	11	2.7	9	2.3		
Strong	28	7	27	6.7		

† Independent *t*-test.

# Fisher's exact test.

* Significant *p*-value of <0.05.

SD, standard deviation.

PA, physical activity.

### The association between PA status and perceived benefits of and barriers to PA

There was a significant difference between physically active and non-physically active participants regarding PA's psychological and social benefits. Physically active patients perceive these benefits more than non-physically active patients. However, no significant difference between the two groups of patients was detected as regards the physical benefits of PA, such as “preventing overweight,” “decreasing the risk of coronary artery disease,” and “decreasing triglycerides.” Improving knowledge about the physiological benefits of PA is an essential element that should be included in the PA programs provided to patients with CHD.

While regarding perceived barriers items about performing PA, physically active and non-physically active participants were significantly different regarding most of the perceived barriers to PA. Physically active patients exhibited fewer physical, psychological, social, and environmental barriers to the perception of PA than non-physically active participants.

### Perceived benefits of and barriers to PA

Most of the benefits perceived by patients with CHD were physiological benefits, such as “improves health” (*M* = 5.81, SD = 0.53) “improves body muscle's flexibility” (*M* = 5.79, SD = 0.52), “improves strength” (*M* = 5.69, SD = 0.58), “prevents overweight” (*M* = 5.74, SD = 0.75), “decreases risk of CHD” (*M* = 5.71, SD = 0.16), “decreases triglycerides” (*M* = 5.70, SD = 0.72), and “improves cardiovascular fitness” (*M* = 5.68, SD = 0.69) (Table 5).

**Table 5:** Benefits of physical activity as perceived by Jordanian patients with CHD.

**Perceived benefits of PA**	**Mean**	**(SD)**	**PA**		**Test**	***P***
			**Yes**	**No**		
1- Improves health	5.81	0.53	5.84	5.04	4.23	0.050^*^
2- Improves flexibility	5.79	0.52	5.82	4.53	5.12	<0.001^*^
3- Prevents overweight	5.74	0.75	5.44	5.13	5.06	0.32
4- Decreases risk of coronary artery disease	5.71	0.16	5.08	5.02	12.32.25	0.17
5- Decreases triglycerides	5.7	0.72	5.88	5.71	4.56	0.18
6- Improves strength	5.69	0.58	5.91	5.14	4.25	0.23
7- Improves cardiovascular fitness	5.68	0.69	4.56	3.97	3.65	0.002
8- Gives enjoyment	5.55	0.77	4.86	3.88	0.58	0.013^*^
9- Keeps the weight from increasing	5.55	0.78	5.23	5.14	0.89	0.15
10- Helps to feel better in general	5.52	0.75	4.36	3.37	1.26	0.023^*^
11- Helps to feel more energetic	5.5	0.77	5.25	4.54	3.58	0.010^*^
12- Prevents hypertension and DM	5.49	0.86	5.12	4.88	4.69	0.020^*^
13- Gives confidence in self	5.44	0.8	4.56	3.25	3.62	0.021^*^
14- Decreases hospital admission	5.43	1.08	4.23	3.983.69	4.89	0.006^*^
15- Improves self-image	5.41	0.84	4.88	4.02	1.58	0.028^*^
16- Provides a way to meet a people	5.4	0.85	4.21	4.25	2.56	0.010^*^
17- Lifts the spirit and causes happiness	5.4	0.84	4.95	3.86	3.58	0.011^*^
18- Improves the appearance	5.39	0.89	3.88	3.1	1.49	0.368
19- Helps to cope better with stress	5.39	0.82	4.13	3.65	2.78	0.002^*^
20- Improves attitude toward life	5.31	0.94	4.63	3.43	3.25	0.001^*^
21- Helps to relax	5.12	1.02	5.02	3.09	2.89	0.002^*^
22- Decreases mortality	4.17	1.66	5.28			0.001^*^

Test: Independent *t*-test.

* Significant *p*-value of <0.05.

SD, standard deviation; PA, physical activity.

The biggest barriers to PA as perceived by the patients were as follows: “feeling with anxiety” (*M* = 4.53, SD = 1.81), “not enough time” (*M* = 4.47, SD = 1.93), “feeling of being too lazy” (*M* = 4.42, SD = 1.81), “busy at work” (*M* = 4.36, SD = 1.86), “lack of interest” (*M* = 4.34, SD = 1.97), “bad weather” (*M* = 4.32, SD = 1.92), “don't like to do PA alone” (*M* = 4.16, SD = 1.98), “not frequently seeing others perform PA” (*M* = 3.93, SD = 2.05), “feeling of being uncomfortable” (*M* = 3.84, SD = 1.76), and “PA causes shortness of breath” (*M* = 3.83, SD = 1.82) (Table 6).

**Table 6:** Barriers to physical activity as perceived by Jordanian patients with CHD.

**Perceived benefits of PA**	**M**	**(SD)**	**PA**		**Test**	***P***
			**Yes**	**No**		
1- Feeling with anxiety	4.53	1.817	4.67	4.33	3.85	0.05^*^
2- Not enough time	4.47	1.936	4.85	4.36	3.98	0.001^*^
3- Feeling like being too lazy	4.42	1.810	4.10	4.96	3.25	0.024^*^
4- Busy at work	4.36	1.861	4.28	4.11	3.54	0.120
5- Lack of interest	4.34	1.971	4.21	4.28	3.55	0.250
6- Bad weather	4.32	1.922	4.02	4.12	3.54	0.250
7- Do does not like to do PA alone	4.16	1.983	3.45	3.52	3.78	0.240
8- Not frequently seeing others doing PA	3.93	2.056	3.85	3.01	3.25	0.002^*^
9- Feeling of being uncomfortable	3.84	1.768	3.92	3.21	3.65	0.002^*^
10- PA causes shortness of breath	3.83	1.826	3.80	3.58	3.88	0.1022
11- Being hot and sweaty	3.76	1.776	3.85	3.74	3.58	0.203
12- No convenient places	3.61	1.999	3.56	3.64	3.21	0.130
13- Fearing of pain in joints or back	3.58	1.810	3.21	3.89	3.52	0.210
14- Feeling too boring	3.57	1.671	3.05	3.55	2.32	0.140
15- Lack of advice	3.57	1.760	3.52	3.25	3.35	0.210
16- PA causes sore muscles	3.51	1.642	3.74	3.45	3.68	0.710
17- Fearing of falling	3.51	1.744	3.42	3.54	3.25	0.410
18- Not seeing friends PA	3.49	1.720	3.65	3.58	3.25	0.280
19- Presence of caregiving duties in the home	3.36	1.851	3.51	3.78	3.52	0.250
20- Lack of confidence	3.34	1.719	3.21	3.41	3.52	0.230
21- Feeling too uncoordinated	3.32	1.614	3.12	3.42	2.85	0.520
22- Having a negative feeling	3.25	1.610	3.10	3.30	2.56	0.120
23- No convenient sidewalks	3.21	1.860	3.18	3.24	2.87	0.410
24- Feeling with depression	3.17	1.551	3.10	3.24	3.74	0.240
25- Family does not encourage doing PA	3.15	1.666	3.12	3.18	3.41	0.210
26- No presence of natural places	3.14	1.886	3.12	3.16	3.54	0.240
27- PA causes dizziness	3.10	1.806	3.8	3.12	3.54	0.180
29- PA causes abnormal BP	2.96	1.699	2.58	2.54	3.21	0.680
30- Fearing of looking silly or funny when the PA	2.75	1.755	2.47	2.54	3.25	0.780
31- Restriction of social norms	2.74	1.790	2.71	2.81	2.58	0.250
32- No presence of enjoyable scenery	2.67	1.793	2.47	2.84	2.74	0.880
33- No presence of street light	2.67	1.531	3.25	2.52	2.54	0.250
34- Having trouble with vision	2.53	1.678	2.54	2.5 8	2.45	0.620
35- Having muscles weakness	2.53	1.306	2.54	2.68	2.41	0.580
36- No feeling safe walking on the street	2.49	1.636	2.37	2.47	2.74	0.250
37- Presence of heavy traffics	2.44	1.540	2.47	2.52	2.84	0.020
38- PA is too expensive	2.37	1.542	2.34	2.40	2.14	0.410
40- PA causes difficulty in sleeping	2.22	1.418	2.18	2.26	2.410	0.170
41- Presence of attended dogs on streets	2.20	1.497	2.14	2.15	2.45	0.160

Test: Independent *t*-test.

* Significant *p*-value of <0.05.

SD, standard deviation; M, Mean; PA, physical activity.

### The influence of sociodemographic and health characteristics on perceived benefits of PA among Jordanian patients with CHD

Job type significantly influenced the perceived benefits of “decreasing triglycerides” and “giving enjoyment.” Laborer workers perceived this PA benefits more than office workers. Physically active participants perceived all physiological benefits more than non-physically active participants.

### The influence of sociodemographic and health characteristics on perceived barriers to PA

This study demonstrated the importance of sociodemographic and health characteristics of patients with CHD in influencing their perception of barriers to PA, including (1) health perception, (2) chest pain, (3) the ability to travel alone, (4) education, (5) age, (6) gender, (7) caring responsibilities, (8) job status, (9) current PA behavior, (10) smoking, and (1) PA advice. Health perception had a significant negative relationship with the barriers “feeling with anxiety” and “PA causes shortness of breath”. Patients with poor health were more likely to perceive “feeling with anxiety” and “PA causes shortness of breath” as solid barriers to PA. Chest pain frequency had a significant positive relationship with “feeling uncomfortable” barriers and “PA causes shortness of breath.” Participants who reported more chest pain frequency were more likely to perceive “feeling uncomfortable” and “PA causes shortness of breath” as solid barriers to PA. Age is negatively related to “busy at work” and “not enough time” barriers. Young participants were more likely to perceive “busy at work” and “not enough time” as solid barriers to PA. No significant relationships with other barriers were found.

Females, illiterate, patients who could not travel alone, smokers, and non-regular physically active perceived “feeling uncomfortable” and “not enough time” as barriers to PA more than their counterparts. Illiterates and individuals who are unable to travel alone perceived “feeling anxiety” as a barrier to PA more than educated individuals who were able to travel alone. Illiterate and non-regular physically active perceived “PA causes shortness of breath” as a barrier to PA more than educated and regular physically active. Individuals who had caring responsibilities perceived being busy at work as a barrier to PA more than those who were not employed and did not have caring responsibilities. Office workers perceived bad weather as a barrier to PA more than laborers workers. PA advice had a significant negative relationship with barriers such as “not enough time,” “feeling of being uncomfortable,” “lack of advice,” and “feeling too boring.”

## Discussion

Although physical activity is widely reported as a principal factor in improving health, it is viewed as a significant challenge for patients with CHD worldwide. This study found that patients were mainly physically inactive (76%). Perceived benefits of and barriers to PA are vital elements influencing PA participation in this study. The physically active patients perceive more benefits of and fewer barriers to PA than non-physically participants. However, no study documented the perceived benefits of and barriers to PA in Jordan among patients with CHD.

All participants in this study reported substantial perceived benefits of PA that were mainly physiological benefits rather than psychosocial benefits, which may be related to the influence of media components that focus on the physiological benefits of PA. Therefore, patients may have more information about the physiological benefits of PA than the psychological benefits of PA. However, physically active patients perceived the physiological and psychosocial benefits of PA more than those who were not regularly physically active. In addition, it may be related that most participants (76%) were not regularly physically active to experience PA's physiological and psychosocial benefits. The role of healthcare providers in reinforcing the knowledge about the impact of physical and psychosocial benefits of PA is a crucial step to be considered in PA programs.

A predominance of perceived psychosocial barriers to PA was observed were “anxiety,” “lack of interest,” “feeling of being too lazy,” “do not like to do PA alone,” and “not frequently seeing others doing PA.” These barriers were also identified in Myers and Roth's studies (26). Anxiety reflects that patients may feel anxious about fear from fatigue or other symptoms when they do PA because they have CHD. “Feeling of being too lazy” reflects that patients have low motivation and negative feelings that inhibit them from PA.

Not enough time was reported in the present study. As such, the need for positive reinforcement and reassurance from healthcare providers who advocate physical activity in CHD is considered necessary to overcome this barrier. The time barrier reflects that individuals are busy at work, which is also perceived as a barrier to PA. It reflects no motivation to do PA because time barriers and busyness at work excuse more than the cause of not participating in PA, which is congruent with the findings of other studies, which showed that time was the most significant barrier perceived by cardiac patients. It is the most prevalent self-reported reason for dropout from supervised clinical and community PA programs (31, 32). Motivation to do PA is predominately prescribed as a barrier to doing PA among adult people (32). The barriers of “lack of interest,” “do not like to do PA alone,” and “not frequently seeing others doing PA” reflect the importance of social support in motivating patients to use PA. It was demonstrated that social support was significantly associated with physical activity behavior (33–35). The physical barriers PA perceived were “feeling of being uncomfortable” and “shortness of breath”. This finding is congruent with the literature, which showed that physical problems were one of the most significant barriers to PA (36–38). Physical barriers that are frequently reported in the literature include fatigue (36), being uncomfortable (37, 38), lacking energy, and shortness of breath (38). After CHD, patients may feel too weak for PA and fear that PA will lead to cardiac events. This feeling may decrease their self-efficacy with PA. Decreased self-efficacy may increase the perception of physical barriers. On the contrary, regular PA could improve self-efficacy to perform PA; thus, the barrier of low self-efficacy may be diminished (39). The result that could be noticed in this study is that the participants who are not physically active perceive a lack of confidence as a barrier to PA more than the physically active participants. The environmental concern of bad weather was strongly perceived as a significant barrier to PA. This finding is congruent with other findings (18). Since some patients have no unique places or closed centers to perform PA as alternatives to brisk outdoor walking, bad weather may be an essential concern. In addition, bad weather seems to reflect no attention and excuses more than barriers to PA.

## Conclusion

This study demonstrated that most patients with CHD (74%) are non-physically active. The challenge of participation in PA was linked to perceived benefits and barriers to PA. The patients in this study perceived various physiological and psychosocial benefits of PA. Also, Jordanian patients with CHD perceived different physical and psychosocial barriers to PA. In addition, it was found that the sociodemographic and health characteristics of the patients with CHD influenced their participation in PA and their perception of PA benefits and barriers to PA. The factors such as age, gender, health perception, chest pain frequency, education, job, PA advice, caring responsibilities, ability to travel alone, smoking, and current PA behavior were considered. It seems that different groups with samples perceived different barriers to PA. The findings of this study suggest that it may be valuable to replicate this study using a more significant and heterogeneous randomly selected sample to increase the generalization of the findings. The findings also suggest investigating barriers to PA as perceived by different groups of people (young, old, female, and educated people) to identify their specific perceived barriers to PA and investigate selected interventions that are appropriate to decrease barriers to PA among patients with CHD. Also, the findings of this study indicate the need for an influential role for nurses and other health team members who provide care to patients with CHD in Jordanian hospitals and clinics. This role should consider the benefits of and barriers to PA and the influence of sociodemographic and health characteristics on perceived barriers to PA in their interventions to motivate the patients to participate in regular PA.

## Data availability statement

The original contributions presented in the study are included in the article/supplementary material, further inquiries can be directed to the corresponding author.

## Ethics statement

The studies involving human participants were reviewed and approved by the Institutional Review Board at King Abdullah University Hospital (KAUH) and Jordan University Hospital Administration. The patients/participants provided their written informed consent to participate in this study.

## Author contributions

EA planned the research, carried out the data collection, analyzed the data, and wrote the manuscript. FB reviewed the paper for publication. All authors contributed to the article and approved the submitted version.
